# Cognitive-Behavioral Coping-Skills Therapy for Alcohol Dependence: Current Status and Future Directions

**Published:** 1999

**Authors:** Richard Longabaugh, Jon Morgenstern

**Affiliations:** Richard Longabaugh, Ed.D., is professor of psychiatry and human behavior and associate director of the Center for Alcohol and Addiction Studies, Brown University, Providence, Rhode Island. Jon Morgenstern, Ph.D., is associate professor of psychiatry and director of Alcohol Treatment and Research Programs, Mount Sinai Medical Center, New York, New York

**Keywords:** cognitive therapy, behavior therapy, coping skills, AODU (alcohol and other drug use) treatment method, AOD (alcohol and other drug) use behavior, treatment outcome, patient-treatment matching, aftercare, combined modality therapy, motivational interviewing, drug therapy, literature review

## Abstract

Cognitive-behavioral coping-skills training (CBST) is an alcoholism treatment approach aimed at improving the patients’ cognitive and behavioral skills for changing their drinking behavior. CBST encompasses a variety of approaches that despite their core similarities differ in duration, modality, content, and treatment setting. Numerous studies and reviews have ranked CBST among the most effective approaches for treating alcoholic patients. Nevertheless, a recent analysis of nine studies failed to identify specific CBST components that could account for the treatment’s effectiveness. Furthermore, a similar analysis of 26 studies suggested that CBST’s superior effectiveness was limited to specific treatment contexts (i.e., when delivered as part of a comprehensive treatment program) and to specific patient subgroups (e.g., patients with less severe alcohol dependence). Several measures may help broaden CBST’s focus and effectiveness, such as incorporating components of other treatment approaches.

The term “cognitive-behavioral coping*-*skills therapy” (CBST) refers to a family of related treatment approaches for alcohol dependence and other psychiatric disorders that aims to treat the patient by improving his or her cognitive and behavioral skills for changing problem behaviors. This article describes the current status of CBST in alcoholism treatment by evaluating CBST’s effectiveness when compared with alternative treatment conditions and by analyzing the mechanisms through which it works. The article also examines whether CBST differs in effectiveness for different kinds of patients, during different treatment phases, or in various potential relapse situations as well as whether any specific CBST approaches are more effective than others. Finally, the article explores the future of CBST and suggests modifications that might enhance the treatment’s effectiveness as well as improve analyses of CBST efficacy.

## Evolution and Definition Of CBST

CBST has its origins in a branch of academic psychology that focuses on understanding how human learning occurs. This approach views any type of psychopathology, including alcohol dependence, as a maladaptive learning process. Accordingly, the central goal of CBST approaches, which exist for numerous psychiatric disorders, is to design techniques through which mal-adaptive responses can be “unlearned” and replaced with adaptive responses. In the early 1970s, social learning theory emerged as a theoretical basis for designing new interventions for people with alcohol problems (Marlatt and Gordon 1985). For example, early studies reported that alcoholic patients who were treated with CBST could be taught to reduce or eliminate their alcohol consumption to a greater extent than could patients who were not treated with CBST (Chaney et al. 1978; Oei and Jackson 1980). Subsequently, research on using CBST in treatment for alcohol problems has been guided primarily by the book *Relapse Prevention: Maintenance Strategies in the Treatment of Addictive Behaviors* by Marlatt and Gordon (1985), which focuses on relapse prevention among patients with alcohol and other drug (AOD) abuse problems (for more information on relapse prevention, see the article in this issue by Larimer and colleagues, pp. 151–160). These studies generally have been conducted by clinical psychologists, often in Veterans Affairs (VA) hospitals.

Over time, CBST has become the alcoholism treatment of choice in academic and VA hospitals. Outside of these settings, however, the Minnesota Model of alcoholism treatment, which is based on the 12-step philosophy of Alcoholics Anonymous, remains the most popular treatment approach. The effectiveness of that approach, however, has not been documented in well-controlled studies—that is, in studies comparing 12-step-treated subjects with control subjects receiving other types of therapy. Thus, a disparity exists between the popularity of a treatment and its demonstrated effectiveness (Hester and Miller 1995).

During the past 25 years, numerous CBST approaches have been developed to treat alcohol dependence; these approaches have differed in many aspects, including duration, modality, content, and treatment setting (Miller et al. 1995). Despite their differences, however, all CBST approaches for alcohol dependence share the following two core elements:

- They espouse the principles of social-cognitive theory (Bandura 1986). As applied to alcohol dependence (Abrams and Niaura 1987), these principles postulate a central role for coping skills. The guiding theory is that deficits in the ability to cope with life stress in general and with alcohol-related stimuli (i.e., alcohol cues) in particular help maintain excessive drinking and lead to a resumption of drinking following aborted attempts at abstinence.
- They employ some form of individual coping-skills training to address the patient’s deficits. For example, each CBST approach teaches skills (using a standard set of techniques) to help the patient identify specific situations in which coping inadequacies typically occur. To enhance the client’s coping skills in those situations, all CBSTs use such teaching tools as instruction, modeling, role play, and behavioral rehearsal.

CBST frequently is classified as a “broad-spectrum treatment approach”—that is, an approach that does not focus primarily on the patient’s alcohol consumption but addresses other life areas that often are functionally related to drinking and relapse. For example, if anger can provoke a patient to drink, the focus of CBST will be on those circumstances that arouse anger in the patient, the thought and behavioral processes that occur between the onset of the anger and the patient’s drinking, and on the events occurring after the patient drinks. Several other broad-spectrum alcoholism treatment approaches exist, including the community-reinforcement approach (CRA), behavioral marital therapy (BMT), behavioral self-control training (BSCT), and relaxation training. The discussion in this article is limited to CBSTs that focus exclusively on coping-skills training of the individual patient, whether this training occurs in group- or individual-therapy sessions. Because BMT, CRA, and BSCT include important therapeutic components other than individual coping-skills training, they have been excluded from this review. These approaches may influence drinking behavior through mechanisms other than those related to coping-skills training. In addition, CRA and BMT both involve other people besides the alcoholic patient in the therapeutic intervention, thereby adding another important element to the therapy. (For more information on CRA, see the article in this issue by Miller and Meyers, pp. 116–121.) Furthermore, in contrast to CBST, which focuses on achieving abstinence, BSCT emphasizes the patient’s choice of a treatment goal (i.e., abstinence or moderate drinking). Finally, relaxation training is excluded from this discussion, because previous reviews have concluded that it is ineffective for alcohol-dependent patients (Miller et al. 1995).

CBST interventions were among the first alcoholism treatment approaches to demonstrate efficacy in reducing drinking in randomized clinical trials^1^ (Chaney et al. 1978; Oei and Jackson 1980). Numerous additional studies during the past 25 years have continued to support CBST’s effectiveness. Moreover, several comprehensive reviews of treatments for alcohol-related problems have ranked CBST approaches among those having the most evidence for clinical and cost effectiveness (e.g., Holder et. al. 1991; Finney and Monahan 1996; Miller et al. 1995). For example, in those reviews, social-skills training was found to be one of the two most effective treatments for alcohol dependence.

### What Are CBST’s Active Ingredients?

Because numerous clinical studies had suggested that CBST was effective in alcoholism treatment (e.g., see Finney and Monahan 1996; Miller et al. 1995), Longabaugh and Morgenstern (1998) reviewed the existing literature to identify mechanisms of action inherent to CBST that contribute to its effectiveness. That is, the investigators attempted to determine which characteristics of CBST were responsible for the fact that alcoholic patients treated with CBST reportedly had better drinking outcomes than did patients treated with various alternative therapies.

The investigators limited the review to well-controlled studies in which the patients had voluntarily entered treatment and had been assigned randomly to receive either CBST or another treatment. Furthermore, patients had to have been either formally diagnosed with alcohol dependence or strongly presumed to be alcohol dependent. Finally, the researchers selected only those studies that attempted to identify the variables responsible for (i.e., the mediators of) CBST’s effectiveness.

To demonstrate that a variable actually mediated CBST effectiveness, the following three factors had to be demonstrated:

- At least part of the observed effectiveness of the treatment (i.e., CBST) had to be attributable to an increase in the mediator variable (e.g., a measure of coping skills).
- A correlation had to exist between the patient’s posttreatment status with respect to the mediator variable (e.g., coping skills) and drinking outcome.
- Statistical analyses had to demonstrate that when the effect of the mediating variable was selectively eliminated, overall treatment effectiveness declined.

Nine studies fulfilled the researchers’ criteria.^2^ The studies measured and analyzed numerous potential mediators for CBST’s effectiveness (e.g., variables measuring coping behaviors and self-efficacy^3^). Only one of the nine studies, however, was able to identify a measure of social skills that attained even marginal status as an actual mediator (Hawkins et al. 1986, 1989). In the remaining studies, either coping skills that increased through CBST were unrelated to drinking outcomes, or coping skills related to drinking outcome were not increased to a greater extent with CBST than with the comparison treatment. Furthermore, several studies did not fully analyze the effects of individual coping skills—that is, the studies did not determine whether CBST increased a particular coping skill more than did the comparison treatment and whether that same skill was related to improved drinking outcome.

In summary, although the review of the nine clinical trials indicated that better coping skills generally were associated with better drinking outcomes, it allowed no conclusions regarding the active ingredients of CBST. The studies demonstrated neither that CBST led to increases in specific coping skills that resulted in better drinking outcomes nor that CBST’s greater effectiveness was attributable to better coping skills. Thus, researchers do not yet know how CBST works to improve drinking outcome. Similarly, recent studies that did not involve random assignment of patients to treatment conditions (i.e., were conducted in naturalistic clinical settings) and which assessed the mechanisms underlying treatment responses also indicated that the active ingredients of CBST are not unique to this approach but are shared by other therapies (e.g., Finney et al. 1998).

### How Effective Is CBST Compared With Other Treatments?

As mentioned previously, several general reviews of treatment effectiveness for patients with alcohol problems have concluded that CBST is one of the most effective interventions. Nevertheless, researchers’ inability to identify the specific mechanisms through which CBST acts (as described in the previous section) suggested a need for a more focused analysis of CBST’s effectiveness. Thus Longabaugh and Morgenstern (1998) identified 26 well-controlled clinical studies published through July 1998; the reports included 39 comparisons of CBST with other treatment approaches.^4^ For example, Project MATCH compared CBST with motivational enhancement therapy and 12-step facilitation therapy (Project MATCH Research Group, 1997*a*). In these 39 comparisons, evidence for CBST’s superior effectiveness depended on two factors:

- The context in which CBST was compared (i.e., whether CBST was delivered as the only treatment, as a component of a more comprehensive treatment, or as aftercare following another treatment)
- The expected effectiveness of the treatment against which CBST was to be compared.

#### Effectiveness of CBST as a Stand-Alone Treatment

Eleven studies compared the effectiveness of CBST delivered as the only (i.e., stand-alone) treatment with the effectiveness of other stand-alone therapies (e.g., supportive group therapy plus naltrexone [O’Malley et al. 1992]). In 10 of those comparisons, CBST was found to be neither more nor less effective than the treatment against which it was compared. In one study, CBST was less effective than the comparison treatment, 12-step facilitation therapy, with respect to the percentage of patients who maintained total abstinence in the year following treatment (Project MATCH 1997*a*). These results indicate that CBST delivered as a stand-alone treatment does not differ in effectiveness from these other treatment approaches.

#### Effectiveness of CBST as Aftercare

CBST also has been delivered as after-care—that is, following completion of a previous, more intensive treatment (e.g., inpatient therapy). In this context, seven studies compared the outcome of patients receiving CBST with the outcome of patients receiving either an alternative treatment or no aftercare. None of those studies found any significant differences in effectiveness between CBST and the comparison treatments (i.e., including no aftercare).

#### Effectiveness of CBST as a Component of a More Comprehensive Therapy

In addition to being a stand-alone treatment, CBST has been included as a component of other treatments, such as inpatient AOD-abuse treatment. A total of 21 studies have evaluated CBST in this context. In 15 of those studies (71 percent), CBST was found to be more effective than the comparison treatment, whereas no differences in effectiveness existed in the remaining 6 studies (29 percent). Thus, patients who receive CBST as a component of a more comprehensive ongoing treatment are likely to have better drinking-related outcomes than patients who do not receive CBST.

##### CBST Effectiveness and Strength of Comparison Treatments

The effectiveness of CBST also can be evaluated by assessing the presumed effectiveness, or “strength,” of the treatment with which CBST was compared. Such analyses have demonstrated that the stronger the alternative therapy was, the less likely CBST was to be more effective than the comparison treatment. For example, when patients receiving CBST were compared with patients receiving no additional therapy, CBST-treated patients had better drinking-related outcomes in 67 percent of the comparisons. When compared with treatments that were likely to be ineffective, such as a general discussion group, CBST was more effective than the comparison treatment in 50 percent of the comparisons and equally effective in the remaining 50 percent of the comparisons. Finally, when CBST was compared with treatments that had a solid theoretical basis (i.e., were theoretically coherent) and therefore could be expected to be effective, CBST was more effective in one comparison (10 percent of comparisons), less effective in another comparison (10 percent), and equally effective in the remaining eight comparisons (80 percent).

Together these analyses suggest that CBST is but one theoretically coherent treatment that can improve the outcome of alcohol-dependent patients. In contrast with previous assessments, the findings further indicate that CBST is more effective than other therapeutic approaches only when added as a component to an ongoing therapy but not when delivered as stand-alone therapy or aftercare. These observations, coupled with the previously reported finding that the mechanisms underlying CBST’s effectiveness remain unknown, indicate that less evidence exists for CBST’s effectiveness than previously believed.

#### CBST and Treatment Matching

Even if CBST is not generally more effective than other therapies in the treatment of alcohol-dependent patients, CBST is still possibly superior to other approaches under certain circumstances. Thus, CBST may be particularly effective during certain treatment phases, in specific high-risk situations, or with patients with certain characteristics. Given CBST’s focus on relapse prevention, it would appear plausible that CBST could be superior to other treatments when used as aftercare therapy, because patients who receive aftercare face day-to-day situations that they may not encounter during a more intensive prior inpatient treatment phase. For example, a patient who is being trained in drink-refusal skills might be more apt to encounter such situations during the aftercare phase of treatment than during primary treatment. As described in the previous section, however, this assumption does not appear to be valid. Similarly, although Marlatt’s classification of relapse situations has provided a framework for designing CBST approaches to improve appropriate coping skills (Marlatt and Gordon 1985), little data suggest that those coping skills are more effective in some high-risk relapse situations than in others. For example, researchers do not yet know whether patients might more effectively resist drinking when they are feeling unhappy than when a friend is offering them a drink. This issue certainly warrants further investigation.

Several studies have attempted to identify patient subgroups that may respond particularly well to CBST. The results of this research, as follows, have been mixed, however, and even contradictory:

- Two studies found that patients with a high degree of sociopathy or with antisocial personality disorder had better drinking outcomes when treated with CBST than when treated with approaches aimed at improving interpersonal relationships (Kadden et al. 1989; Longabaugh et al. 1994). More recent studies, however, have failed to confirm this association or matching effect (Project MATCH 1997*a*, b*b*; Kalman et al. 1996).
- Some researchers have hypothesized that patients with deficits in social skills would be most likely to benefit from CBST (Kadden et al. 1992). Studies investigating this hypothesis have yielded mixed results, however, and two recent studies even suggest the opposite relationship. According to those studies, patients with low problem-solving skills (Jaffe et al. 1996) or greater alcohol-related social dysfunction (Longabaugh et al. 1999) were less likely to benefit from CBST than from more socially supportive treatment approaches. For example, Longabaugh and colleagues (1999) found that patients with lower social-functioning skills had better drinking outcomes when treated with 12-step facilitation therapy, a treatment aimed at involving patients in Alcoholics Anonymous (AA), a mutual self-help group, than when treated with CBST.
- Investigators also have hypothesized that patients with more severe psychiatric dysfunction would respond better to CBST than to treatments that do not focus on psychiatric impairment (Kadden et al. 1989). Again, the results of studies assessing this proposition are contradictory (Kadden et al. 1989; Project MATCH 1997*a*,*b*).
- For two patient characteristics that have been thought to be associated with lowered rates of CBST success—cognitive impairment and greater alcohol dependence—clinical studies also have yielded equivocal results. For example, conflicting evidence exists as to whether mildly to moderately cognitively impaired patients benefit less from CBST than from alternative treatments (Jaffe et al. 1996; Project MATCH 1997*a*,*b*). Similarly, evidence suggesting that patients with different severity of alcohol dependence will respond differently to various treatment approaches is also mixed. Most recently, Project MATCH found that among patients exhibiting fewer symptoms of alcohol dependence, those treated with CBST in an aftercare setting had better treatment outcomes than those treated with a 12-step program. Conversely, among patients exhibiting more symptoms of alcohol dependence, those who received CBST had worse outcomes than those who received 12-step-oriented therapy (Project MATCH 1997*b*).

In summary, although some evidence supports the hypothesis that CBST efficacy differs among various patient subtypes, the results of relevant studies are mixed and the extent of any observed association generally is small. Consequently, more studies and stronger evidence will be needed before one can draw the conclusion that CBST is more effective than other treatment approaches for specific patient subgroups.

## Future Directions for Research

Several factors may contribute to the apparent lack of difference in effectiveness between CBST and other theoretically coherent treatment alternatives and may explain the differences in outcome compared with earlier studies. First, alternative treatments may have improved in efficacy over time. Second, as a result of changes in other treatment approaches, CBST may no longer be as distinctive as it used to be. For example, studies of the active ingredients of CBST have suggested that patients receiving alternative treatments (e.g., 12-step therapy) use the same mechanisms of change as do CBST patients (Finney et al. 1998). Both patients receiving CBST and those receiving 12-step facilitation therapy, for example, are less likely to spend time in settings that used to be associated with drinking. Furthermore, some researchers have incorporated CBST-based relapse-prevention strategies into 12-step programs (Gorski and Miller 1982).

Third, the studies that have tested CBST’s effectiveness and mechanisms of action may not have shown complete fidelity to the tenets of CBST’s conceptual framework. For example, one key element of CBST is a detailed analysis of each patient’s drinking pattern to identify individual antecedents and consequences of drinking. The results of this functional analysis provide the basis for developing that patient’s specific treatment plan. Such a plan can indicate which situations the patient should avoid, how the patient should deal with those situations if they do occur, and which alternative behaviors (other than drinking) the patient should use to cope with problematic situations. However, few research studies of CBST’s effectiveness—and none of the studies reviewed in the previous sections—have included individualized functional analyses to guide the treatment of individual patients. Instead, therapies used in those studies have relied on teaching social skills for dealing with commonly occurring problematic situations.

Another tenet of CBST is that the skills to be learned should be mastered and applied in real-life situations before the therapist can conclude that the patient has adequately acquired the skills. Again, however, such demonstrations generally have not been a component of CBST research studies. Consequently, some patients in those studies may have never mastered the necessary skills, thereby reducing the effectiveness of CBST. The number of treatment sessions in CBST research studies generally averages about 12. It is possible that this number is too low for CBST to develop its full effect.

### Broadening CBST’s Focus

Assuming, however, that the CBST approaches tested in research studies adequately reflect the treatment administered in real-life clinical settings and that the reported results therefore accurately represent CBST’s effectiveness, the findings reported in this article clearly demonstrate that CBST’s effectiveness could be increased appreciably. One approach to doing so could be to incorporate components of other effective therapies, just as other therapies have broadened their focus and enhanced their effectiveness by including CBST strategies. Several possibilities exist for this approach.

First, motivational interviewing—a strategy aimed at increasing the patient’s motivation for change—has been found to increase the effectiveness of other treatment approaches (e.g., Brown and Miller 1993). As commonly delivered, CBST assumes that the patient already is motivated to stop or reduce drinking and that he or she only needs to acquire the skills to do so. This assumption, however, may not always be correct, and some patients may have the appropriate coping skills but lack the motivation to use them. For those patients, the incorporation of motivational interviewing into CBST could increase skill use.

Second, research has consistently shown that patient involvement in self-help groups, such as AA, is associated with positive drinking outcomes (e.g., Tonigan et al. 1996). CBST could easily incorporate a referral module to increase the likelihood that patients become involved in mutual self-help groups. The incorporation of such an element into the CBST treatment plan may potentiate CBST effectiveness, particularly if it involves self-help groups with belief systems similar to those underlying CBST (e.g., SMART Recovery [1995]). In SMART, the underlying assumption, like in CBST, is that alcohol consumption is at least in part a learned mal-adaptive behavior that the person has within himself or herself the power to change. In contrast, an AA belief is that the individual is powerless to change his or her drinking without the help of a “higher power.” Consequently, patients treated with CBST who are referred to AA may experience difficulty in the shift in belief system about what it takes to get better.

Third, several studies have demonstrated the effectiveness of therapeutic approaches that aim to incorporate one or more significant others into the treatment of people with alcohol problems, such as BMT (O’Farrell 1989) and CRA (Meyers and Smith 1995). Similar to CBST, these therapies seek to modify the ways in which a patient responds to a stimulus to drink and the consequences that the patient will experience depending on whether or not he or she does take a drink. By modifying the patient’s response, the patient will be more likely to maintain sobriety in order to achieve well-being. In contrast with CBST, however, BMT and CRA enlist significant others from the patient’s social environment directly into the treatment; thus, the quality of the patient’s relationships with those significant others can be enhanced and made contingent on sobriety (Longabaugh et al. 1995). The incorporation of environmental factors into CBST also may increase therapeutic control over the reinforcing factors that can help a patient maintain an alcohol-free lifestyle.

Fourth, CBST might easily incorporate treatment strategies based on classical conditioning procedures, such as cue exposure (Monti et al. 1993). Classical conditioning posits that stimuli or cues that repeatedly have co-occurred with drinking (e.g., the sight of a bar or the smell of alcohol) eventually can elicit craving for alcohol and precipitate drinking. During cue exposure, patients are directly exposed to alcohol-related stimuli (e.g., an alcoholic beverage) and taught skills to cope with these cues. The repeated exposure to alcohol cues is thought to eliminate, or extinguish, the previous responses to those cues (i.e., craving and drinking). (For more information on cue-exposure therapy, see the article in this issue by Monti and Rohsenow, pp. 107–115.)

Fifth, although current CBST therapies generally focus on teaching skills for coping with situations with high risk for relapse, early studies of CBST’s effectiveness also frequently included general social-skills training. This approach was based on the assumption that if a patient lacks general skills for coping with life’s demands, failures in coping may lead to general unhappiness. Unhappy people, in turn, may not be motivated to deprive themselves of the transient reduction in distress that may follow alcohol consumption. Consequently, current CBST therapies might be improved by refocusing, at least in part, on assisting patients to learn general skills for coping with life.

Finally, in addition to incorporating elements of other psychosocial therapy approaches, CBST also might benefit from the addition of pharmacotherapies. For example, the medication acamprosate^5^ has been found to be useful in delaying relapse (Litten and Allen 1991), and the medication naltrexone has been shown to reduce heavy drinking following a relapse (e.g., O’Malley et al. 1992). The addition of such pharmaceutical agents to CBST might provide patients with a buffer against urges to reinitiate drinking or drink to excess, thereby expanding the patient’s opportunity to learn the skills that can help avoid a relapse.

## A Next-Generation, Broad-Spectrum Behavioral Treatment

As suggested in the preceding section, the focus and effectiveness of CBST could be broadened by incorporating important ingredients from other broad-spectrum behavioral treatments and from therapies that have not arisen from, but are compatible with, social learning theory (e.g., self-help groups and pharmacotherapies). Given all these suggestions for adding components to CBST and for intensifying CBST approaches, the question arises: How could such extensive treatment be provided to all alcohol-dependent patients? The answer is that most likely such an all-encompassing treatment approach would not be necessary for each patient, because most patients would not need to be exposed to each of the treatment components. Instead, each patient could be matched to the specific treatment elements that he or she needs.

Along those lines, several matching studies have attempted to link specific patient characteristics to different treatment requirements. For example, in the previously mentioned Project MATCH study, patients were randomly assigned to one of three standardized and largely uniformly delivered treatments—CBST, motivational enhancement therapy, and 12-step facilitation therapy—in order to identify patient characteristics that would allow selection of the most appropriate therapy. In contrast, this article proposes that a single treatment be developed that allows for selecting specific components of one comprehensive approach to match each patient’s preference or need. Such a matching process could be conducted by using decision trees that triage patients through a menu of options based on their stated preferences or assessed needs. For example, a patient who feels a greater need to learn better communication skills might select this module instead of a module that focused on mood management.

Two such treatment modalities are currently being developed for implementation with alcohol-dependent patients. One of these modalities, which combines pharmacotherapy with behavioral therapy, is being studied in Project COMBINE, a multicenter randomized clinical trial being conducted by the National Institute on Alcohol Abuse and Alcoholism in collaboration with 11 universities. In this study, the behavioral therapy component combines motivational interviewing, referral to mutual self-help groups, and involvement of a supportive significant other, and selection of coping-skills modules based on patient preference. With that approach, patients first develop self-change plans based on assessments of the feedback they have received regarding the effects of their drinking on their lives. Subsequently, patients select the appropriate options from a menu of coping-skills modules to facilitate execution of their self-change plans.

The second example of a CBST approach that is driven by decision trees based on assessed patient strengths and deficits is called Broad Spectrum Therapy, a methodology developed by Gulliver and Longabaugh (Longabaugh 1999). In this approach, a systematic assessment of the patient’s strengths and deficits becomes the basis for designing an individualized treatment plan (see figure below). Depending on the emergent patient profile, different modules within the menu of treatment options are incorporated into the treatment plan. Furthermore, the choice of treatment modules can be reevaluated and adjusted as the patient’s alcohol-related problems change during treatment.

**Figure:**
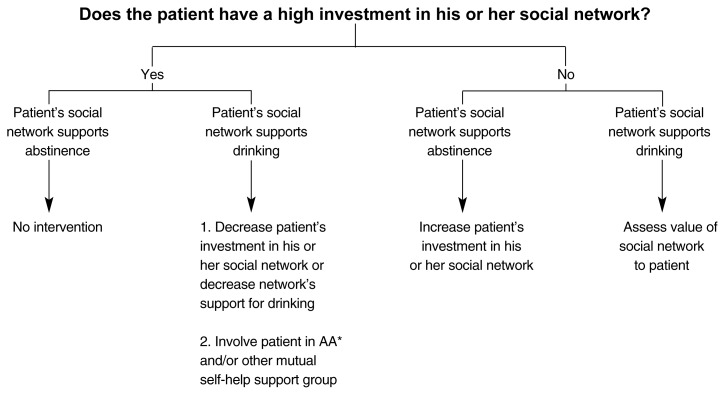
An example of a decision tree for assessing a patient’s social network. The therapist first assesses how highly invested the patient is in his or her social network (i.e., How many people are in the patient’s social network? How much time does the patient spend with them? Does the patient regard the members of his or her social network as important?). Next, the therapist evaluates whether the network supports the patient’s drinking or abstinence. Network members who support drinking frequently drink themselves, drink a lot per drinking occasion, and encourage or accept the patient’s drinking. Finally, the therapist determines the patient’s treatment based on the assessment of this information. *AA = Alcoholics Anonymous.

Both the treatment delivered in Project COMBINE and Broad Spectrum Therapy are, of course, not unlike CBST delivered by many competent therapists in everyday clinical practice. Thus, individualized treatment plans are developed and implemented for many patients in response to patient preference and therapist assessment of need. In clinical research, however, treatment generally has been standardized so that all patients are offered more or less the same treatment because of researchers’ fears that results obtained with less uniform protocols cannot be generalized outside of the research setting. The authors of this article propose, however, that in order to obtain results that can be generalized, it may not be necessary to deliver the same standard treatment to all patients but to apply the same decision trees to all patients. If uniform decision trees are used, the selection of treatment modules based on those decision trees will be consistent, and the resulting treatments can be replicated and generalized to everyday clinical practice.

Clinical research testing the effectiveness of such an approach to treatment will evaluate the efficacy of the underlying principles of the treatment rather than the implementation of the specific package selected by each individual patient. Thus, study results would tell researchers whether using decision trees as a generic guiding principle improves patient outcomes. Such studies would provide less information, however, on whether specific alternatives chosen by patients using each of these decision trees are helpful. Consequently, the primary focus of the evaluation would be the efficacy of the underlying treatment principles rather than the efficacy and implementation of specific treatment components or individual treatment plans. Such an approach to clinical research should more accurately assess treatment effectiveness (i.e., how well the therapy works in the everyday world of delivery of care) as well as efficacy (i.e., whether or not a treatment can work under highly controlled experimental conditions). Therapies developed using this approach may improve the effectiveness currently achievable by standard CBST treatment protocols.

## Footnotes

1 Randomized clinical trials are studies in which patients are randomly assigned to different treatments. In this case, the participants received CBST or another intervention.

2 A complete list of references for those nine studies is available from the authors of this article.

3 In this context, self-efficacy refers to the patient’s belief that he or she can successfully deal with situations likely to lead to drinking.

4 A complete list of the references for the studies included in this analysis is available from the authors of this article.

5 Acamprosate has not yet been approved for commercial use in the United States.
